# The modulatory effect of electrical stimulation on the excitability of the corticospinal tract varies according to the type of muscle contraction being performed

**DOI:** 10.3389/fnhum.2014.00835

**Published:** 2014-10-16

**Authors:** Kei Saito, Kenichi Sugawara, Shota Miyaguchi, Takuya Matsumoto, Hikari Kirimoto, Hiroyuki Tamaki, Hideaki Onishi

**Affiliations:** ^1^Department of Physical Therapy, Niigata University of Health and WelfareNiigata, Japan; ^2^Institute for Human Movement and Medical Sciences, Niigata University of Health and WelfareNiigata, Japan; ^3^Faculty of Rehabilitation, Kanagawa University of Human ServicesKanagawa ,Japan

**Keywords:** electrical stimulation, voluntary contraction, excitability, corticospinal tract, transcranial magnetic stimulation

## Abstract

Afferent input caused by electrical stimulation of a peripheral nerve increases corticospinal excitability during voluntary contractions, indicating that proprioceptive sensory input arriving at the cortex plays a fundamental role in modulating corticospinal excitability. The purpose of this study was to investigate whether the effect of electrical stimulation on the corticospinal excitability varies according to the type of muscle contraction being performed. Motor-evoked potentials (MEPs) were elicited by transcranial magnetic stimulation (TMS) during a shortening contraction, an isometric contraction, or no contraction of the first dorsal interosseous (FDI) muscle. In some trials, electrical stimulation of the ulnar nerve was performed at 110% of the sensory threshold or 110% of the motor threshold prior to TMS. Electrical stimulation involved either a train of 50 pulses at 10 Hz or a single pulse. Shortening contraction with the train of electrical stimuli significantly increased MEP amplitudes, and the increase was dependent on the type of stimulation. Isometric contraction with the train of electrical stimuli and electrical stimulation without voluntary contraction did not affect MEP amplitudes. A single pulse of electrical stimulation did not affect MEP amplitudes in any condition. Thus, electrical-stimulation-induced modulation of corticospinal excitability varied according to the type of muscle contraction performed and the type of stimulation. These results show that the type of contraction should be considered when using electrical stimulation for rehabilitation in patients with central nervous system lesions.

## INTRODUCTION

Application of electrical stimulation concurrent with voluntary movement facilitates motor recovery (Bolton et al., 2004; de Kroon et al., 2005; McDonnell et al., 2007; Hara et al., 2013) and induces substantial cortical reorganization in patients with central nervous system injuries such as stroke (Kimberley et al., 2004; Bhatt et al., 2007; Fujiwara et al., 2009; Hara et al., 2013). A neuroimaging study using transcranial magnetic stimulation (TMS) indicated that electrical stimulation with voluntary movement resulted in increased corticospinal excitability concomitantly with functional motor recovery in stroke patients (Tarkka et al., 2011). Thus, increasing corticospinal excitability is considered to be involved in the mechanism underlying functional motor recovery from stroke using this combined therapeutic approach.

The stimulation intensity was effective in modulating the change in corticospinal excitability by electrical stimulation with voluntary movement. When the stimulated muscle is voluntarily shortened, low-intensity electrical stimulation displays significantly increased corticospinal excitability than high-intensity electrical stimulation (Saito et al., 2013). However, the effect of stimulation intensity on corticospinal excitability is considered to depend on whether or not the stimulated muscle is active. Chipchase et al. (2011b) reported that the stimulation intensity is strongly associated with the modulation of corticospinal excitability when electrical stimulation is delivered without voluntary movement. Khaslavskaia et al. (2002) showed that electrical stimulation increases the excitability of the corticospinal projection to the stimulated muscle in an intensity-dependent manner when the stimulated muscle is quiescent.

Voluntary movement differs in its ability to modulate the corticospinal excitability in response to the type of muscle contraction being performed. A previous study in monkeys showed that somatosensory input evoked by voluntary movement is quantitatively different between the types of muscle contraction (Seki and Fetz, 2012). Lee and White (1974) reported that shortening contractions increased the somatosensory evoked potential (SEP) greater than did isometric contractions. These studies indicate that somatosensory input by shortening contraction is quantitatively larger than input by isometric contraction. Together with the observation that shortening contractions increased corticospinal excitability significantly more than did isometric contraction (Chye et al., 2010), movement related sensory gating is considered to be associated with modulation of corticospinal excitability.

The synergistic effect of afferent input by electrical stimulation and somatosensory input by voluntary movement is known to play an important role in modulating corticospinal excitability (Kido Thompson and Stein, 2004; Khaslavskaia and Sinkjaer, 2005; Barsi et al., 2008; Saito et al., 2013; Sugawara et al., 2013). However, it remains unknown whether the effect of stimulation intensity on corticospinal excitability is dependent on the type of muscle contraction being performed. The somatosensory and electrical afferent inputs both differ with the type of muscle contraction and stimulation intensity; thus, it is important to clarify the optimal stimulation intensity to effectively increase corticospinal excitability for a given type of muscle contraction combined with electrical stimulation. Thus, we examined the influence of stimulation intensity on the change in corticospinal excitability by electrical stimulation in response to the type of voluntary movement. The results of this study may indicate the appropriate intensity of electrical stimulation for treatment of motor dysfunction using voluntary movement concurrent with electrical stimulation.

The purpose of this study was to elucidate the effect of electrical stimulation of the ulnar nerve on the excitability of the corticospinal projection to the first dorsal interosseous (FDI) muscle during voluntary shortening contractions and voluntary isometric contractions.

## MATERIALS AND METHODS

### PARTICIPANTS

Fourteen neurologically normal right-handed volunteers (thirteen males and one female) with a mean age of 22.1 ± 1.7 years participated in this study. All volunteers provided written informed consent before participation. This study was performed in accordance with the Declaration of Helsinki, and the protocol was approved by the Research Ethics Committee of Niigata University of Health and Welfare.

### ELECTROMYOGRAM RECORDING

During all experiments, the participant sat comfortably in a chair and placed his or her right hand on a table with the palm perpendicular to the horizontal plane. Surface electromyograms were recorded from the right FDI muscle using disposable silver–silver chloride surface electrodes (N-00-S; Mets Inc., Tokyo, Japan).

### ELECTRICAL STIMULATION OF THE ULNAR NERVE

Electrical nerve stimulation was applied to the right wrist to stimulate the ulnar nerve that innervates the FDI muscle. Stimulation was generated using an electrical generator (SEN-7203; Nihon Kohden Co., Tokyo, Japan) with an isolator (SS-104; Nihon Kohden Co.) and a pair of silver-silver chloride surface electrodes. Stimulation was delivered in trains of 50 pulses at 10 Hz with a pulse width of 1 ms. Two different stimulus intensities were used: (i) 110% of the motor threshold (above motor threshold) and (ii) 110% of the sensory threshold (above sensory threshold). The motor threshold was defined as the lowest stimulus that evoked a visible twitching of the index finger, and the sensory threshold was defined as the lowest stimulus that the volunteer could perceive. In this study, the average motor threshold was 11.2 ± 4.7 mA (mean ± SD), and the average sensory threshold was 7.4 ± 2.7 mA.

### VOLUNTARY HAND MOVEMENT TASKS

**FIGURE 1:**
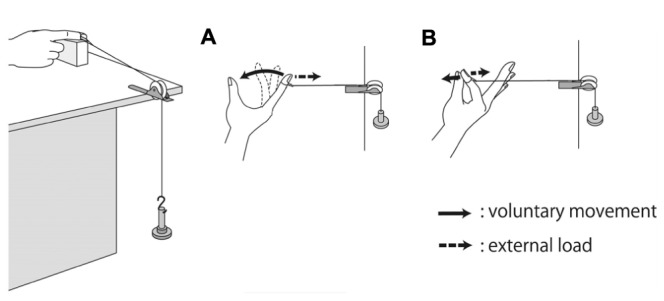
**Experimental set-up and voluntary hand movement. (A)** For the shortening contraction task, the participant was required to flex the metacarpophalangeal (MP) joint of the index finger from 0 to 90° and to touch the index finger to the thumb. **(B)** For the isometric contraction, the volunteer was required to maintain the MP joint of the index finger at 90° against external extension.

The volunteers were asked to perform the following hand movement tasks: (i) shortening contraction and (ii) isometric contraction (**Figure 1**) to investigate how the influence of electrical stimulation on the excitability of the corticospinal tract was altered by the difference in somatosensory input induced by voluntary hand movement. In the shortening contraction task, the volunteer flexed the metacarpophalangeal (MP) joint of the index finger from 0 to 90° while the index finger received external torque from a custom-made apparatus that connected distal interphalangeal (DIP) joint of the index finger to a scale weight via a pulley to easily sustain constant activity of FDI muscle (**Figure 1**). The shortening contraction was a single movement which was held for 5 s. In the isometric contraction task, the volunteer performed an isometric pinch movement, attempting to touch the index finger to the thumb. The index finger was passively separated from the thumb by the same custom-made apparatus (**Figure 1**), and the volunteer was required to maintain the MP joint of the index finger at 90° for the 5-sec duration of the task. In both hand movements, the volunteer set the EMG of the FDI muscle to 15% MVC on the basis of visual EMG feedback during voluntary hand movement; this procedure was practiced until reaching an EMG of 15%MVC.

### TMS

Transcranial magnetic stimulation was delivered over the left primary motor cortex using a figure-eight coil with an internal wing diameter of 90 mm connected to a Magstim 200 (Magstim Co. Ltd., Whitland, UK). The optimal site for eliciting motor-evoked potentials (MEPs) from the FDI (motor hotspot) was found by delivering a slightly suprathreshold stimulus at 1-cm intervals around the assumed motor hotspot. The coil was placed tangentially to the scalp and held at 45° to the midsagittal line. The TMS intensity was set to 120% of the resting motor threshold for tasks that involved no voluntary contraction and 120% of the active motor threshold for tasks that involved voluntary contraction. The resting motor threshold was defined as the minimum stimulus intensity that evoked a MEP greater than 50 μV in at least five out of ten trials. The active motor threshold was defined as the minimum stimulus intensity that evoked a small MEP (> 100 μV) in at least five out of ten trials when the volunteer made the FDI muscle contract isometrically, and made the EMG of FDI muscle 5%MVC with visual EMG feedback.

### EXPERIMENTAL PROCEDURE

The volunteers performed the following experimental tasks in a randomly assigned order (**Table 1**): (i) shortening contraction with electrical stimulation; (ii) shortening contraction without electrical stimulation; (iii) isometric contraction with electrical stimulation; (iv) isometric contraction without electrical stimulation; (v) electrical stimulation without voluntary contraction. Electrical stimulation included the following two different stimulus conditions: (i) a train of electrical stimuli in which the stimulus duration was set to 5 s; (ii) single electrical pulse just before TMS with the following two different intensities: (i) above motor threshold; (ii) above sensory threshold. A single electrical stimulus just before TMS influences MEPs evoked by TMS in response to the interstimulus interval of a single electrical pulse and a TMS pulse, i.e., short-latency afferent inhibition and afferent facilitation (Mariorenzi et al., 1991; Deletis et al., 1992; Chen et al., 1999; Tokimura et al., 2000; Roy and Gorassini, 2008; Russmann et al., 2009; Devanne et al., 2009), so that a single electrical pulse just before TMS may influence the MEP changes induced by a train of electrical stimuli. Thus, this study investigated not only MEP changes induced by a train of electrical stimuli but also changes induced by a single electrical pulse just before TMS. A total of 14 experimental tasks were performed successively on the same day. For the combination of voluntary hand movement and electrical stimulation, electrical stimulation began to be delivered along with voluntary hand movement and was switched off after 5 s. When a single electrical pulse was combined with voluntary hand movement, electrical stimulation was only delivered just before the TMS trigger.

**Table 1:** Experimental study design.

Voluntary contraction	Electrical stimulation (ES)
Shortening contraction	With a train of ES (above motor threshold)
Isometric contraction	With a train of ES (above sensory threshold)
	With single electrical pulse (above motor threshold)
	With single electrical pulse (above sensory threshold)
	Without ES

At rest	A train of ES (above motor threshold)
	A train of ES (above sensory threshold)
	Single electrical pulse (above motor threshold)
	Single electrical pulse (above sensory threshold)

**FIGURE 2:**
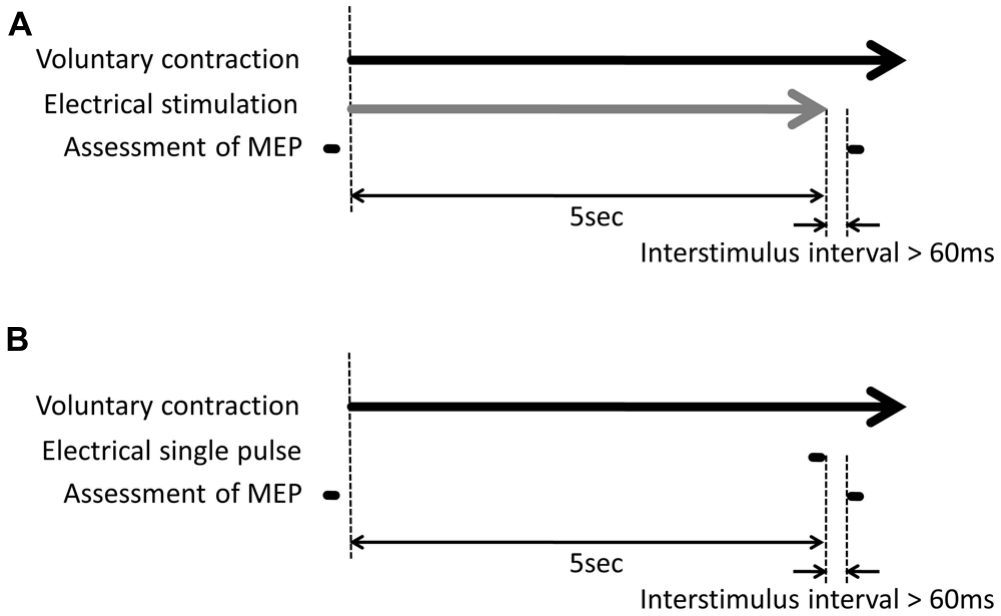
**Experimental protocol. (A)** For the combination of voluntary hand movement and electrical stimulation, electrical stimulation began to be delivered along with voluntary hand movement and was switched off after 5 s. Transcranial magnetic stimulation (TMS) measurement was made before and after the experimental task. The TMS trigger was delivered while the volunteers performed the voluntary hand movement. The TMS trigger was delivered 60 ms after the last electrical pulse. **(B)** For the combination of voluntary hand movement and a single electrical pulse, the volunteers performed the voluntary hand movement without electrical stimulation. Electrical stimulation was only delivered 60 ms before the TMS trigger.

Transcranial magnetic stimulation measurements were made before and just after the experimental tasks (**Figure 2**). In TMS measurements before the experimental task, TMS was delivered with 120% of both the resting and active motor thresholds to measure control MEPs. In TMS measurements after the experimental task, TMS was delivered while the volunteers performed voluntary hand movements. The TMS trigger was applied 60 ms after the last electrical stimulus pulse to minimize the influence of the electrical pulse on the MEPs and detect the effect of electrical stimulation on the MEPs.

### DATA ANALYSIS

For voluntary contraction with electrical stimulation, the peak-to-peak MEP amplitude was measured and expressed relative to the peak-to-peak MEP amplitude observed in the control trial (MEP control ratio). For electrical stimulation without voluntary contraction, the peak-to-peak MEP amplitude was measured. The root mean square (RMS) amplitude of the FDI electromyogram was calculated in the 50 ms prior to the TMS trigger and expressed relative to the RMS amplitude observed with isometric maximum FDI contraction (RMS maximum ratio). A two-way repeated measured analysis of variance (ANOVA) with factors of muscle contraction (isometric contraction or shortening contraction) and type of electrical stimulation (with a train of electrical stimulation above motor threshold, with a train of electrical stimulation above sensory threshold, with single pulse above motor threshold, with single pulse above sensory threshold or without electrical stimulation) was used to analyze the different effect of electrical stimulation on the corticospinal excitability among the type of muscle contraction and to analyze the background EMG among each task. For each type of contraction, a repeated-measures ANOVA with the type of contraction as one factor was used to compare the MEP control ratio and the mean RMS maximum ratio across experimental tasks. *Post hoc* testing was performed using the Tukey multiple comparison. For each type of electrical stimulation, Student’s paired *t*-test (two-tailed) was used to compare the MEP control ratio between isometric contraction and shortening contraction. Further, for each stimulus condition of electrical stimulation, a repeated-measures ANOVA with the stimulus condition of electrical stimulation as one factor was used to compare the peak-to-peak MEP amplitude. All statistical analyses were conducted using SPSS 15.0 for Windows. Statistical significance was determined as *p* < 0.05 for all comparisons.

## RESULTS

### ELECTRICAL STIMULATION-INDUCED MODULATION OF CORTICOSPINAL EXCITABILITY

A two-way repeated-measures ANOVA revealed significant effect of the voluntary hand movement [*F*(1.13) = 19.638, *p* = 0.001] and type of electrical stimulation [*F*(4.52) = 4.191, *p* = 0.023] on the corticospinal excitability. Furthermore, the analysis also revealed significant interaction between voluntary hand movement and the type of electrical stimulation [*F*(4.52) = 2.956, *p* = 0.028].

#### Shortening contraction task

**FIGURE 3:**
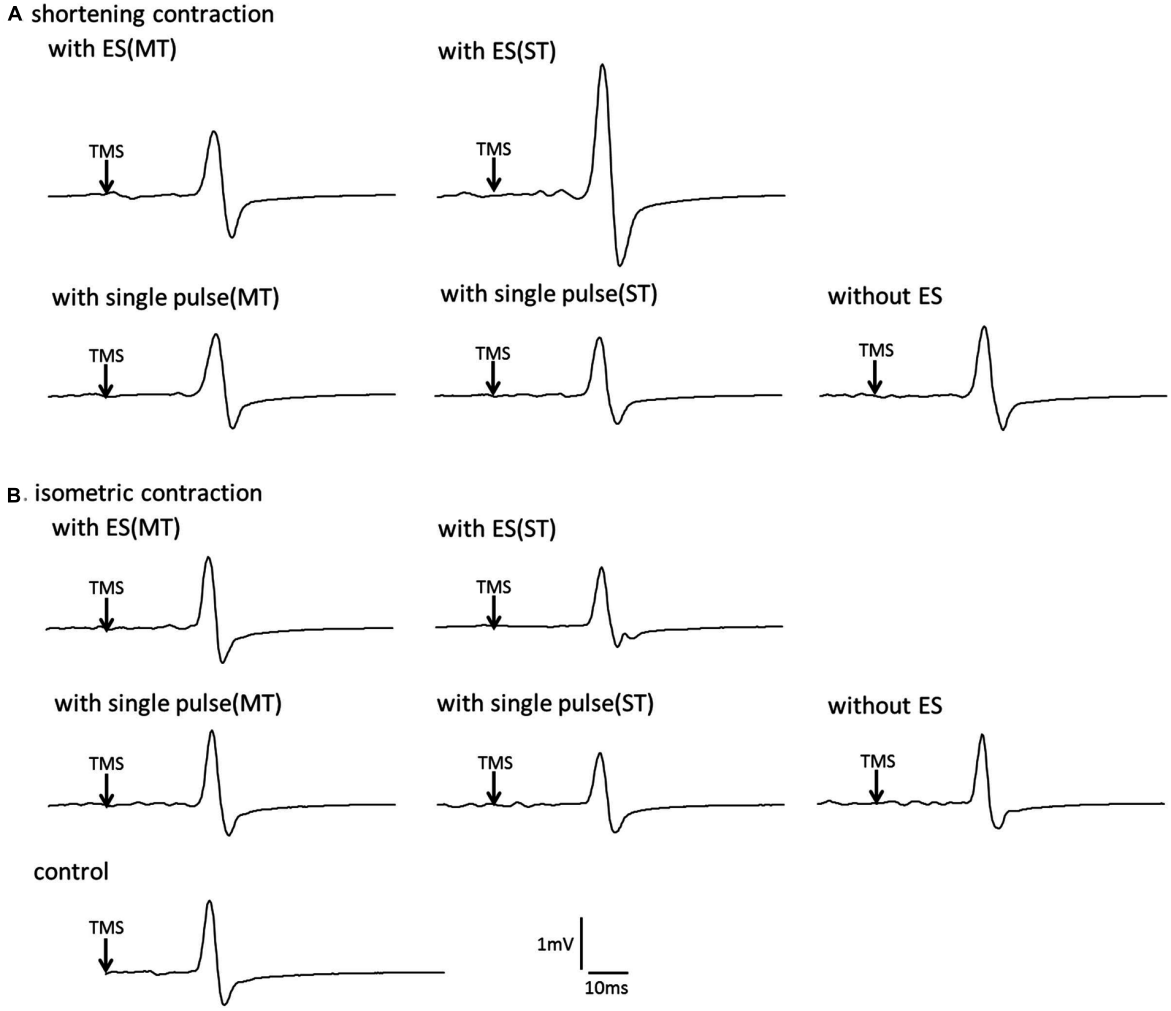
**The effect of voluntary contraction with electrical stimulation on the corticospinal excitability in single subject **(A)** Typical motor-evoked potential (MEP) waveforms recorded in the first dorsal interosseous (FDI) muscle. (A)**, shortening contraction. **(B)**, isometric contraction.

**FIGURE 4:**
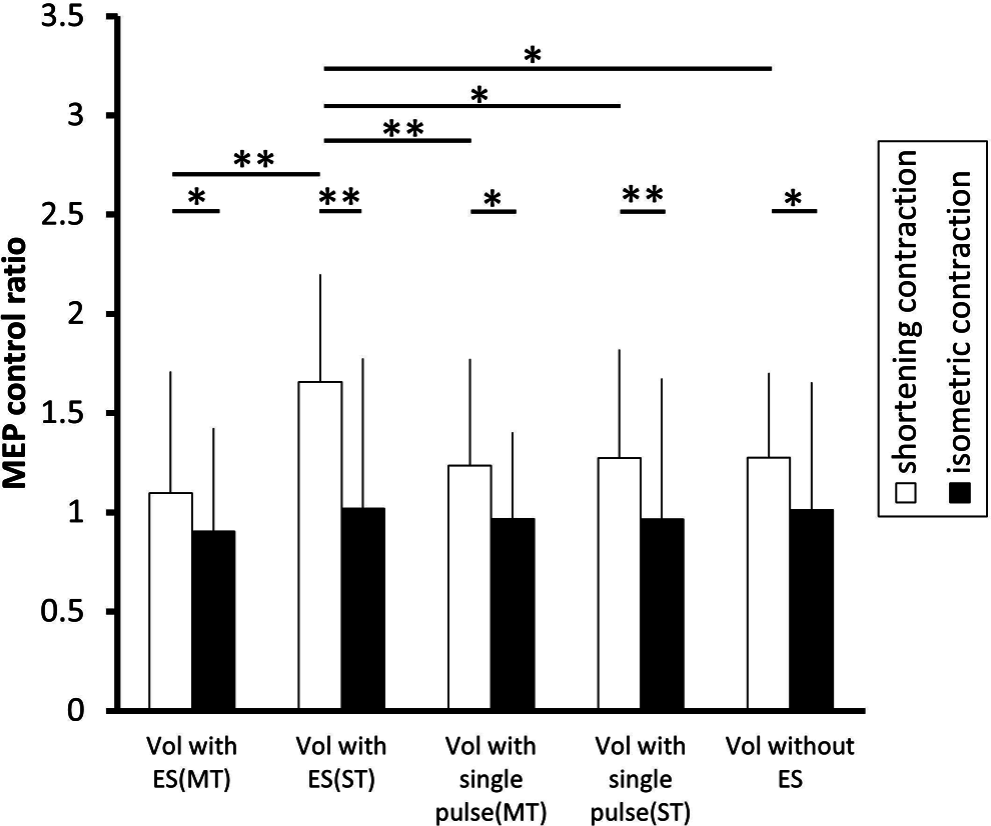
**The effect of voluntary contraction with electrical stimulation on the excitability of the corticospinal tract.** The changes in the group mean MEP control ratio (*n* = 14) induced by the following experimental tasks: voluntary contraction (vol) with electrical stimulation (ES; above motor threshold, MT), vol with ES (above sensory threshold, ST), vol with a single electrical pulse (MT), vol with a single electrical pulse (ST), and vol without ES. In shortening contraction (white bar), the MEP control ratio was significantly highest when a voluntary shortening contraction was coupled with ES (ST) than when a voluntary shortening contraction was coupled with other types of electrical stimulation (all *p* < 0.05 ). In isometric contraction (black bar), the MEP control ratio was similar across experimental tasks [*F*(4.52) = 0.600, *p* = 0.665]. Further, the MEP control ratio was significantly higher during shortening contraction than isometric contraction in all type of electrical stimulation (all *p* < 0.05). *p< 0.05, **p< 0.01. Error bars indicate standard deviation (SD).

**Figure 3A** shows typical MEP waveforms in the FDI muscle from one participant recorded in a shortening FDI contraction performed with and without a single pulse of ulnar nerve stimulation or a train of ulnar nerve stimuli, and **Figure 4** shows the pooled data (*n* = 14). A one-way repeated-measures ANOVA revealed a significant effect of the type of electrical stimulation on the MEP control ratio [*F*(4.52) = 5.002, *p* = 0.002 ]. *Post hoc* analysis revealed that the MEP control ratio during shortening contraction was significantly higher with a train of electrical stimulation above sensory threshold than with other types of electrical stimulation (*p* < 0.05). However, the analysis also revealed no significant difference between the MEP control ratio during shortening contraction with electrical stimulation above motor threshold and that without electrical stimulation (*p* = 0.658). These results indicated that a train of low-intensity electrical stimulation was highly effective in facilitating MEPs measured in the FDI during the shortening contraction rather than high-intensity electrical stimulation.

#### An isometric contraction task

**Figure 3B** shows typical MEP waveforms in the FDI muscle from one participant during an isometric contraction performed with and without a single pulse of ulnar nerve stimulation or a train of ulnar nerve stimuli, and **Figure 4** shows the pooled data (*n* = 14). A one-way repeated-measures ANOVA revealed that the MEP control ratio was similar across experimental tasks [*F*(4.52) = 0.600, *p* = 0.665], indicating that ulnar nerve stimulation did not affect the MEPs measured in the FDI during isometric FDI muscle contraction.

#### The corticospinal excitability and muscle contraction being performed

A Student’s paired *t*-test revealed that the MEP control ratio was significantly higher during shortening contraction than that during isometric contraction in all types of electrical stimulation (*p* < 0.05), indicating that shortening contraction displayed higher MEPs measured in FDI than isometric contraction regardless of stimulation type.

#### Electrical stimulation without voluntary contraction

**FIGURE 5:**
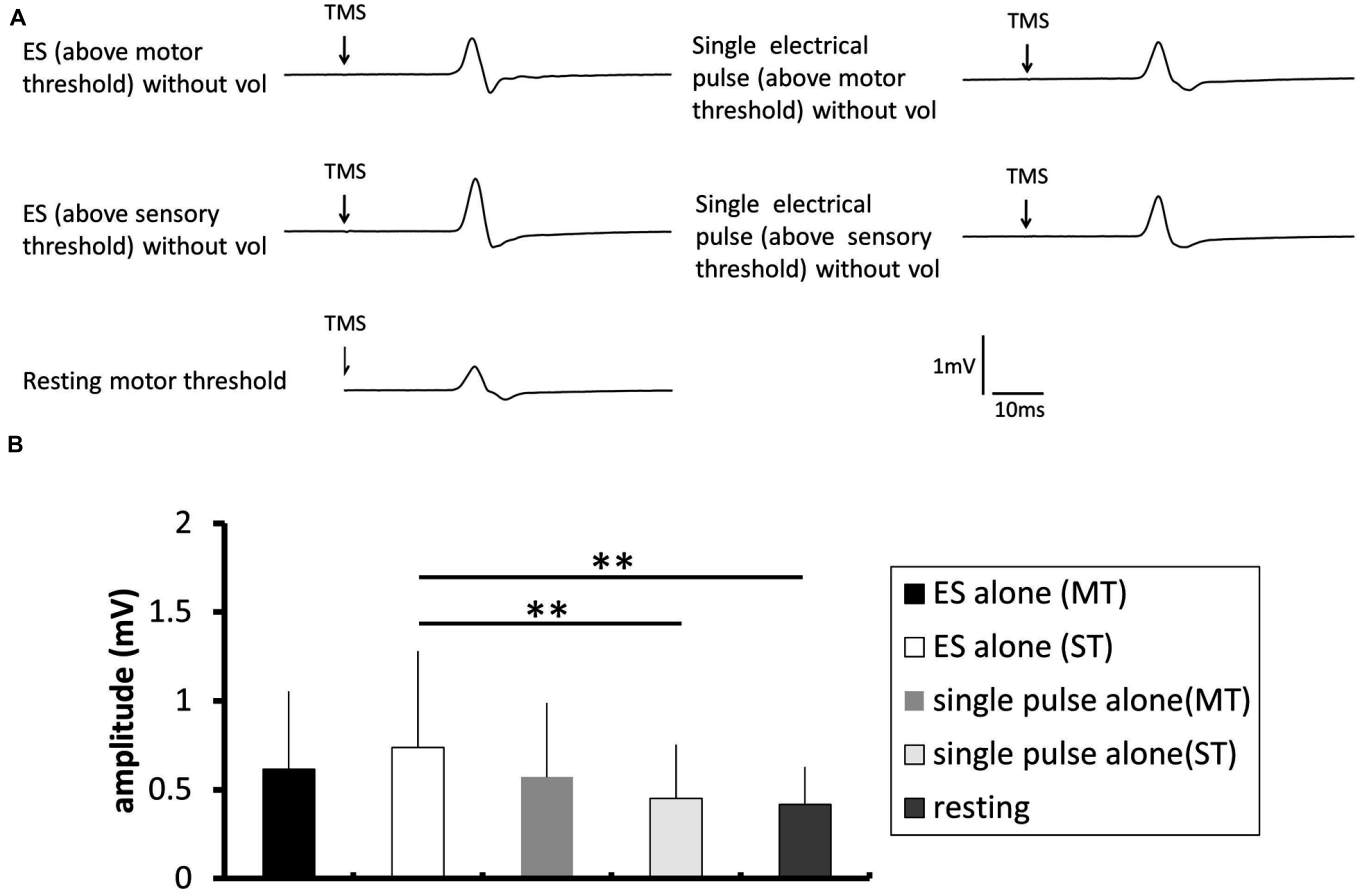
**The effect of electrical stimulation without voluntary contraction on the excitability of the corticospinal tract. (A)** Typical averaged motor-evoked potential (MEP) waveforms recorded in the FDI muscle. **(B)** The changes in the group mean MEP control ratio (*n* = 8) induced by the following experimental tasks: electrical stimulation (ES; above motor threshold, MT) without voluntary contraction (vol), ES (above sensory threshold, ST) without vol, single electrical pulse (MT) without vol, and single electrical pulse (ST) without vol, and a control trial using the resting motor threshold as the TMS intensity. The MEP amplitude was significantly higher after a train of electrical stimulation above sensory threshold than after single pulse above sensory threshold (*p* = 0.018) and at rest (*p* = 0.006).

**Figure 5A** shows a typical MEP in the FDI from one participant during a train of electrical stimulation of the ulnar nerve without voluntary contraction, and **Figure 5B** shows the pooled data (*n* = 14). A one-way repeated-measures ANOVA revealed that there was a significant effect of the stimulation condition on the MEP control ratio [*F*(4.52) = 4.201, *p* = 0.005]. *Post hoc* analysis revealed that the MEP amplitude was significantly higher after a train of electrical stimulation above sensory threshold than that after a single pulse above sensory threshold (*p* = 0.018) and that at rest (*p* = 0.006).

### RMS ELECTROMYOGRAPHY

**Table 2** shows the average RMS maximum ratio of the FDI electromyography signal in the 50 ms prior to the TMS trigger. A two-way repeated-measures ANOVA revealed no significant effect of the voluntary hand movement [*F*(1.13) = 1.004, *p* = 0.335] and type of electrical stimulation [*F*(4.52) = 1.929, *p* = 0.181] on the backgroung EMG. Furthermore, the analysis revealed no significant interaction between voluntary hand movement and the type of electrical stimulation [*F*(4.52) = 1.733, *p* = 0.157].

**Table 2:** Background activity of the first dorsal interosseous (FDI) muscle during each task.

	The type of voluntary contraction		
	Shortening contraction	Isometric contraction
Vol with ES (above motor threshold)	0.16 ± 0.04	0.16 ± 0.03
Vol with ES (above sensory threshold)	0.14 ± 0.05	0.13 ± 0.04
Vol with single electrical pulse (above motor threshold)	0.16 ± 0.08	0.14 ± 0.08
Vol with single electrical pulse (above sensory threshold)	0.14 ± 0.04	0.14 ± 0.04
Vol without ES	0.13 ± 0.06	0.13 ± 0.05

*Average ± SD*

## DISCUSSION

The main finding from these experiments was that a train of electrical stimuli of the ulnar nerve during voluntary hand movement modulated the excitability of the corticospinal projection to the FDI, and that the modulation was dependent on the pattern of muscle contraction. In shortening contraction, low-intensity electrical stimulation displayed increased corticospinal excitability compared with high-intensity stimulation. In isometric contraction, a train of electrical stimulation did not affect the excitability of the corticospinal tract. On the other hand, in resting condition, low-intensity electrical stimulation was effective for increasing the corticospinal excitability.

### THE EFFECT OF SHORTENING CONTRACTION WITH ELECTRICAL STIMULATION ON THE CORTICOSPINAL EXCITABILITY

During the shortening contraction task, low-intensity electrical nerve stimulation increased the excitability of the corticospinal tract, but high-intensity electrical nerve stimulation did not. This is consistent with the results found in our previous study, in which the effect of electrical stimulation on corticospinal excitability was dependent on stimulus intensity (Saito et al., 2013).

The additive effect of increased corticospinal excitability by shortening contraction, plus increased excitability by electrical stimulation, may explain the additional increase in corticospinal excitability during shortening contraction combined with low-intensity electrical stimulation. A previous study reported that corticospinal excitability is highly increased during a shortening contraction (Kasai et al., 1997). Furthermore, our study also showed that low-intensity electrical stimulation displayed a significantly increased corticospinal excitability when the stimulated muscle was at rest. This is consistent with the observations of previous studies showing that electrical stimulation increases corticospinal excitability (Ridding et al., 2000, 2001; Kaelin-Lang et al., 2002; Khaslavskaia et al., 2002; Mckay et al., 2002; Charlton et al., 2003; Knash et al., 2003; Tinazzi et al., 2005; Mang et al., 2010, 2011; Chipchase et al., 2011a,b; Golaszewski et al., 2012; Andrews et al., 2013). The combination of electrical stimulation with voluntary contraction yielded the greatest increase in corticospinal excitability (Khaslavskaia and Sinkjaer, 2005), suggesting these inputs may induce activation of multiple pyramidal cells, leading to a state of subliminal fringe. Consequently, we believe the TMS pulse was able to activate more pyramidal cells during a shortening contraction with low-intensity electrical stimulation.

In contrast, the additive effect might be specific for low-intensity electrical stimulation with shortening contraction. This study showed that high-intensity electrical stimulation was not effective for modulating the corticospinal excitability during shortening contraction. Considering that high-intensity electrical stimulation did not affect corticospinal excitability when the stimulated muscle was at rest, afferent input by high-intensity electrical stimulation might be insufficient to modulate corticospinal excitability. Thus, the additive effect might not be induced, even when shortening contraction is combined with high-intensity electrical stimulation.

### THE EFFECT OF ISOMETRIC CONTRACTION WITH ULNAR NERVE STIMULATION ON THE CORTICOSPINAL EXCITABILITY

Electrical stimulation of the ulnar nerve did not affect the corticospinal excitability when the volunteer was maintaining a constant index finger MP joint angle while exerting a constant torque against a rigid restraint. This is not consistent with the observation that low-intensity electrical stimulation but not high-intensity electrical stimulation is effective in increasing corticospinal excitability. These results indicated that isometric contraction might result in a loss of the low-intensity electrical stimulation-induced effect on the corticospinal excitability.

This discrepancy might be due to the movement-related gating of the sensory input. Previous studies have demonstrated gating of sensory input during voluntary contraction (Angel and Malenka, 1982; Blakemore et al., 1998; Bays et al., 2005), indicating attenuation of afferent input during the contraction. Thus, in this study, afferent input by electrical stimulation might be reduced by sensory gating during isometric contraction. Furthermore, the duration of electrical nerve stimulation in the present study (5 s), was shorter than that used in previous studies, where electrical stimulation increased the corticospinal excitability (Ridding et al., 2000, 2001; Mang et al., 2011; Golaszewski et al., 2012; Léonard et al., 2013). Thus, the combined use of isometric contraction and electrical stimulation may not have been sufficient to increase corticospinal excitability.

### THE CORTICOSPINAL EXCITABILITY AND MUSCLE CONTRACTION BEING PERFORMED

The corticospinal excitability was significantly higher during shortening contraction than that during isometric contraction regardless of the intensity and type of electrical stimulation.

The different level of movement-related sensory gating between the muscle contractions being performed is believed to be responsible for this result. Previous studies have demonstrated gating of sensory input during voluntary contraction (Angel and Malenka, 1982; Blakemore et al., 1998; Bays et al., 2005). Another study showed that a shortening contraction increased the SEP over that of isometric contraction (Lee and White, 1974). These results indicate the level of somatosensory processing that induces attenuation of somatosensory input is stronger during isometric contraction than during shortening contraction. Thus, in this study, the level of sensory gating during isometric contraction with electrical stimulation is likely to be stronger than that during shortening contraction with electrical stimulation.

### THE EFFECT OF ULNAR NERVE STIMULATION WITHOUT VOLUNTARY CONTRACTION ON THE CORTICOSPINAL EXCITABILITY

Low-intensity electrical stimulation was effective in increasing the corticospinal excitability, but high-intensity electrical stimulation was not effective. These results indicated that the effect of electrical stimulation on corticospinal excitability was specific for the stimulation intensity. This is not consistent with the observation from the previous studie, where the increase in the corticospinal excitability elicited by electrical stimulation was reported to be dependent on stimulus intensity (Khaslavskaia et al., 2002).

This discrepancy might be due to sensory gating induced by electrical muscle contraction. A previous study reported that gating of sensory input was present during passive movement as well as during voluntary movement (Jones et al., 1989). High-intensity electrical stimulation used in this study caused muscle contraction without voluntary effort and consequently might have induced the gating of sensory input as well as voluntary contraction. Along with the short duration of electrical nerve stimulation in the present study (5 s), high-intensity electrical stimulation that has been used here might be insufficient to increase the corticospinal excitability.

### METHODOLOGICAL CONSIDERATIONS

An increase or decrease in the excitability of spinal interneurons may be involved in an increase in the excitability of the corticospinal tract to the FDI muscle. Christova et al. (2010) reported that muscle vibration that externally stimulates the muscle spindle does not regulate the excitability of the spinal cord during a voluntary contraction. Furthermore, electrical stimulation does not affect the excitability of the spinal cord (Thompson et al., 2006). Thus, electrical stimulation-induced modulation of corticospinal excitability during voluntary contraction may be predominantly caused by a change in the excitability of the primary motor cortex. Further examination of the mechanism underlying the changes in corticospinal excitability induced by the combination of voluntary contraction and electrical stimulation is necessary.

## CONCLUSION

Our results suggest that electrical nerve stimulation-induced modulation of corticospinal excitability is dependent on the type of voluntary muscle contraction being performed. This indicates that the intensity of electrical stimulation should be set according to the hand motor task being performed when a combination of voluntary contraction and electrical stimulation is utilized for rehabilitation of patients with central nervous system lesions.

## AUTHOR CONTRIBUTIONS

Kei Saito, Kenichi Sugawara, and Hideaki Onishi. Performed the experiments: Kei Saito, Shota Miyaguchi, Takuya Matsumoto, and Hideaki Onishi. Analyzed the data: Kei Saito, Hideaki Onishi, Hikari Kirimoto, Hiroyuki Tamaki, and Kenichi Sugawara. Wrote the paper: Kei Saito, Hideaki Onishi, Kenichi Sugawara, Hikari Kirimoto, and Hiroyuki Tamaki.

## Conflict of Interest Statement

The authors declare that the research was conducted in the absence of any commercial or financial relationships that could be construed as a potential conflict of interest.
